# Best Practices for Building Interprofessional Telehealth: Report of a Conference

**DOI:** 10.5195/ijt.2021.6434

**Published:** 2021-12-16

**Authors:** Lynda B. Ransdell, M. Elizabeth Greenberg, Emi Isaki, Alan Lee, Janet P. Bettger, Goris Hung, Amy Gelatt, Ambur Lindstrom-Mette, Jana Cason

**Affiliations:** 1 College of Health and Human Sciences, Northern Illinois University, Dekalb, Illinois, USA; 2 Northern Arizona University, Flagstaff, Arizona, USA; 3 Department of Physical Therapy, Mount Saint Mary's University, Los Angeles, California, USA; 4 Department of Orthopaedic Surgery, Duke University, School of Medicine, Durham, North Carolina, USA; 5 Hong Kong Polytechnic University, Kowloon, Hong Kong; 6 University of Arizona, College of Nursing, Tucson, Arizona, USA; 7 Auerbach School of Occupational Therapy, Spalding University, Louisville, Kentucky, USA

**Keywords:** Future implications, Nursing, Occupational therapy, Physical therapy, Speech-language pathology, Telemedicine, Telepractice

## Abstract

The Arizona Biomedical Research Centre (ABRC) has funded a series of workshops and conferences since 2016 to build the capacity of local, tribal, and state agencies, healthcare delivery organizations, and non-governmental organizations to engage in meaningful research related to health disparities. With the COVID-19 pandemic, the use of telehealth has dramatically increased, particularly in nursing, occupational therapy (OT), physical therapy (PT), and speech-language pathology (SLP). The purpose of this paper is to summarize the presentations and discussion from the conference titled “Telerehabilitation and Telepractice: An Interprofessional Conference to Build Connections and Best Practices,” held remotely on March 4-5, 2021. Terminology and concepts from the conference were debated, modified, and refined, based on an interprofessional audience. Presenters at the conference, all leaders in their field, discussed the current status of telehealth in their professions, including best practices, challenges, future trends, and research needs.

This introduction is designed to introduce the reader to: (a) a general history of telehealth, (b) the evolution and growth of telehealth, (c) the rationale and definitions for various terms used to refer to telecommunication as applied to the health professions; (d) modes of telehealth delivery; and (e) general fundamentals and best practices for effective telehealth.

## GENERAL HISTORY OF TELEHEALTH

Telehealth incorporates various health-related services from multiple disciplines. In regions where it was available and convenient, telehealth became the ideal solution at the height of the pandemic for keeping clients at home but continuing their care. COVID-19 has challenged healthcare like no other health problem in modern times. Because of the rapid spread of the virus, the possibility of significant and long-term health conditions, and the risk of variants, along with advances in technology and practice, the use of telehealth has expanded rapidly. The benefits of telehealth coupled with the now widespread acceptance of telehealth in general suggest telehealth is here to stay.

### GENERAL EVOLUTION & SUCCESS OF TELEHEALTH

Telehealth has grown considerably in scope and use in recent years thanks to advances in and availability of technology. To ensure safety and positive outcomes, clinicians have a responsibility to keep pace with the rapid growth and potential of telehealth. To date, the majority of telehealth professionals learn their roles and responsibilities on the job.

The success of telehealth depends on clinician and client competence with the technology, and communication and collaboration with the client and among the disciplines involved. When these competencies, communication, and collaboration are in place, telehealth benefits clients and providers by reducing gaps in care and increasing convenience and access to much needed services otherwise unavailable. It provides clients with the benefits of being treated in their own home, saving time, money, and the need for transportation, and assuring safety from exposure during the pandemic.

### TERMINOLOGY

This section describes the use of various forms of telecommunications by clinicians to deliver rehabilitation services, or consultations with other clinicians who are located at a distance (i.e., they are not “in-person” in the same physical space). For the purposes of this paper, unless the intervention is specific to medical treatment, persons who receive services will be referred to as “clients,” a term that is used by most of the disciplines participating in this paper, and a more general and inclusive term than “patient,” and rehabilitation professionals (i.e., practitioners) will be referred to as “clinicians.”

The nomenclature employed to describe this relatively new and evolving service delivery method is not yet consistent among the rehabilitation professions. The use of different terms by professions to describe remote service delivery via telecommunications is possibly reflective of the still maturing nature of tele-therapy, as well as how various professions perceive their professional roles and services.

Given the reference to “medicine” in the association's name, it is no surprise that the American Telemedicine Association (ATA), as an early and influential proponent of diagnosis, treatment, and consultation at a distance adopted the term “telemedicine,” and embraced even more descriptive terminology of sub-specialties, (i.e., tele-dermatology; tele-intensive care unit (ICU); tele-nursing and telerehabilitation). The American Telemedicine Association (ATA) has recently shifted to increasingly referring to “telehealth” on its website (American Telemedicine Association, 2020).

When referring to remote service deliver by speech-language pathologists (SLPs), “telepractice” (not “telehealth”) is the term explicitly approved for use by the American Speech-Language-Hearing Association (ASHA) (American Speech-Language Hearing Association, n.d.). ASHA prefers “telepractice” over “telehealth” because SLPs practice in educational settings as well as in medical and other health related settings, and SLPs treat a wide range of communication disorders that are not necessarily related to health conditions.

In contrast, the American Occupational Therapy Association (“Telehealth in Occupational Therapy,” 2018) advocates for use of the term “telehealth,” as does the American Physical Therapy Association (American Physical Therapy Association, 2019). Such inconsistencies in terminology are not optimal, especially when different professions serve one individual and deliver coordinated services on an interprofessional team. Differences in terminology reinforce professional silos; inconsistent terminology can be disruptive for team-based care and limit the impact of efforts across professions in legal, policy, marketing, and reimbursement reform; and even require awkward choices to be made when team members from different professions author articles or grants together.

Unless specifically referring to SLP practice (i.e., telepractice), this paper broadly uses the term “telehealth” to denote remote service delivery via telecommunication by nurses, occupational therapists, physical therapists, and speech-language pathologists. In some cases, where rehabilitation is the focus, telerehabilitation will be used.

The nomenclature that denotes elements of time and space in clinical practice is also currently in flux:

1. *Asynchronous telehealth* occurs when the client and the clinician do not simultaneously engage in communication. Examples include: *“store and forward”* clinical data that is recorded and then forwarded later to a practitioner, videos/images, e-mails, and other stored electronic content. Asynchronous telehealth can also include data from wearable medical devices or even data from pressure sensors that is received by the clinician, but not in real-time.
2. *Synchronous telehealth* occurs when the client and clinician are both present and communicating at the same time. This typically occurs by a platform such as videoconferencing that provides both visual and audio communication. Synchronous telehealth can also be inclusive of sensors that transmit data in “real time” to the practitioner at the same time s/he and the client are engaged in a session. Phone or wireless communications that only present “audio” content can be described as synchronous but convey less data than technologies that simultaneously convey both visual and auditory information.
3. *Hybrid telehealth* methods suggest some combination of time and space approaches. One common understanding of a “hybrid” approach is when a client is treated both in-person, and at other times, at a distance. Another use of the “hybrid” terminology occurs when some combination of synchronous and asynchronous telecommunication methods is used (e.g., e-mailed instructions and video conferencing.)
4. *In-person* is a phrase that is in the process of replacing “face-to-face” to denote communication that occurs at the same time, in the same physical space. That is because “face-to-face” communication can occur either in-person, or via a video session, wherein both the practitioner and the client can view each other's faces, in real-time (Cason, 2017).

### GENERAL FUNDAMENTALS AND BEST PRACTICES OF TELEHEALTH

Telehealth is in a constant state of growth and advancement. Best practices are those that help meet and improve client outcomes and are supported by evidence. Because of the constant changes in technologies and services, professionals have a responsibility to identify, learn, and share best practices for telehealth.

#### INTERPROFESSIONAL TEAM BEST PRACTICES

Evidence clearly shows that interprofessional practice (IPP) is most successful when team members have experienced interprofessional education (IPE). However, like telehealth education, IPE is not often included in formal education programs.

Clients in need of rehabilitation often require services from multiple disciplines. Therapeutic goals, interventions, and progress will vary depending on individual needs and the corresponding discipline. Communication and collaboration among team members to coordinate client care is a standard of interprofessional practice.

#### FUNDAMENTAL BEST PRACTICES FOR TELEHEALTH AND TELEREHABILITATION

Richmond and colleagues convened a working group of the American Telemedicine Association's Special Interest Group on Telerehabilitation and published a paper on best practices for telerehabilitation (Richmond et al., 2017). The following is a summary of selected fundamental best practices that apply to telehealth across the rehabilitation professions.

1. Abide by federal, state, and regional laws, including licensure and certification requirements, and lawful reimbursement (Brannon et al., 2012; Cohn & Cason, 2019).
2. Foster equitable interprofessional collaboration to the benefit of the client (Cohn & Cason, 2019; World Federation of Occupational Therapists, 2014).
3. Obtain client or family consent via an explicit, informed consent form for telehealth (Cohn & Cason, 2019).
4. Select a technology that is accessible for the client and allows for HIPAA compliant practice (Watzlaf & Ondich, 2012).
5. Establish a Business Associate Agreement (BAA) to ensure HIPAA compliance when using a third party's technology (HHS.gov, 2013; Watzlaf & Ondich, 2012).
6. Communicate to your client and his/her family, if applicable, a plan to manage internet interruptions and other technology failures (Watzlaf & Ondich, 2012).
7. Ensure client privacy and confidentiality (Cohn & Cason, 2019).
8. Identify, train, and supervise appropriate e-helpers or family members that can assist before, during, and after a session, if applicable (Douglass et al., 2021).
9. Establish client safety protocols that include a knowledge of where the client is located for each session, persons that can be called upon to assist in an emergency, and phones of emergency services that are specific to the client's location (Cohn & Cason, 2019).
10. Abide by ethical professional practices (Cohn & Cason, 2019).

In addition to the aforementioned principles of best practice, several resources are available from various professional associations (See Table 1). These resources tend to be discipline-specific, so continued work should be done to facilitate interprofessional education and practice.

**Table 1:** Recommended Resources and Practices for Telehealth

Discipline	Organization & Web Contents	Web Address
Nursing	American Academy of Ambulatory Care Nursing	https://www.aaacn.org/practice-resources/telehealth
Occupational Therapy	American Occupational Therapy Association: Telehealth Resources	https://www.aota.org/Practice/Manage/telehealth.aspx
Physical Therapy	American Physical Therapy Association, Telehealth in Practice	https://www.apta.org/your-practice/practice-models-and-settings/telehealth-practice
SpeechLanguage Pathology	American Speech-Language-Hearing Association, Telepractice Portal	https://www.asha.org/practice-portal/professional-issues/telepractice/
Multiple Professions	American Congress of Rehabilitation Medicine (ACRM)	https://acrm.org/rehabilitation-medicine/rehabilitation-medicine-telemedicine-strategies/
	American Telemedicine Association (ATA)	https://www.americantelemed.org
	Center for Connected Health Policy (CCHP)	https://www.cchpca.org/
	Arizona Telemedicine Program	https://telemedicine.arizona.edu
Multiple Professions	National Consortium of Telehealth Resource Centers, “a collaborative of 12 regional and 2 national Telehealth Resource Centers (TRCs), committed to implementing telehealth programs for rural and underserved communities. Funded by the U.S. Department of Health and Human Services (HHS) Health Resources and Services Administration (HRSA), administered through grant # G22RH30365, TRCs across the nation provide timely and accurate information on telehealth.”	https://telehealthresourcecenter.org/
	Telehealth Code of Ethics	https://www.americanboardoftelehealth.org/wp-content/uploads/2021/03/CORE_Curiculum_Ethics_Infographic_20-ECAR-22207.pdf
Multiple Professions	*International Journal of Telerehabilitation* (an open source, subscription-free biannual journal)	http://telerehab.pitt.edu/ojs/index.php/telerehab
	Interprofessional Education Collaborative	https:/ipec.memberclicks.net

## WORKSHOP PLANNING

The Arizona Biomedical Research Centre (ABRC) has funded a series of workshops and conferences since 2016 to build the capacity of local, tribal, and state agencies, healthcare delivery organizations, and non-governmental organizations to engage in meaningful research related to health disparities. With the COVID-19 pandemic, the use of telehealth has dramatically increased, particularly in nursing, occupational therapy (OT), physical therapy (PT), and speech-language pathology (SLP). The use of interprofessional practice via telehealth is emerging, therefore, the purpose of this paper was to summarize the presentations and discussion from the conference titled “Telerehabilitation and Telepractice: An Interprofessional Conference to Build Connections and Best Practices,” held remotely on March 4-5, 2021. Terminology and concepts from the conference were debated, modified, and refined, based on an interprofessional audience. Presenters at the conference, all leaders in their field, discussed the current status of telehealth in their professions, including best practices, challenges, future trends, and research needs.

The workshop was conceived and organized by the principal investigators (Ransdell and Trotter) and the Program Manager (Gelatt) of the ABRC grant. The speakers were recruited by the first author, based on their reputations in their respective field, and their participation in professional associations as experts in interprofessional telehealth. The workshop agenda and content were collaboratively developed by the speakers, who met several times on Zoom to discuss the contents and format. The workshop was divided into five sections with a goal to promote information sharing and small group discussions via breakout rooms. Speakers agreed to maximize dissemination of information from the conference by publishing this paper.

## WORKSHOP OVERVIEW

A summary of the speakers and topics is presented in Table 2. The first day featured six sessions. The first session of the conference started with an overview of telehealth, with a focus on telepractice (i.e., tele-speech and tele-audiology). Topics for this session, led by Dr. Ellen Cohn, included evaluation, assessment, monitoring, prevention, intervention, supervision, education, consultation and coaching across multiple settings. Procedures for conducting safe and private telepractice were shared. The second session, led by Dr. Emi Isaki, focused on clinical experiences and best practices for speech-language pathologists. Additionally, requirements to provide telepractice services in Arizona, consideration of ethical issues, and a review of interprofessional practice were presented

**Table 2:** Summary of Speakers and Topics (See: https://nau.edu/cher/past-events/)

Speaker & Discipline	Expertise	Topic & Learning Objectives (where applicable and CEUs assigned)
Ellen Cohn, PhD, CCC-SLP, F-ASHA	Faculty member at University of Pittsburgh; founding Editor of *International Journal of Telerehabilitation;* founding Coordinator of ASHA's Special Interest Group on Telepractice; former ATA Board member and coordinator of the ATA Telerehabilitation SIG, and co-author of books on tele-AAC, telerehabilitation, communication disorders and communication.	“An Introduction to Telepractice in 2021” Learning Objectives: Define telepractice List the potential benefits of telepractice Describe a procedure to conduct safe telepractice Identify a privacy concern relevant to telepractice Show an ASHA web-based resource dedicated to telepractice
Emi Isaki, PhD, CCC- SLP	Professor at Northern Arizona University; Research interests include mild TBI, early cognitive-communication screening and therapy, communication related to community re-entry following stroke and TBI, telerehabilitation, and multicultural communication issues.	“Telepractice in 2021: The Arizona Clinical Experience and Best Practices” Learning Objectives: Summarize licensure requirements for telepractice in AZ Describe strengths and challenges in SLP telerehabilitation Identify ethical issues in the provision of telerehabilitation Discuss the implications of interprofessional practice using telerehabilitation
Alan Lee, PT, PhD, DPT	Professor at Mount Saint Mary's University in Los Angeles. Served as the Secretary of Telerehabilitation SIG for the American Telemedicine Association; currently Vice President of Technology SIG for HPA; telehealth group leader for the American Physical Therapy Association's Frontiers in Rehabilitation, Science and Technology Council.	“Advancement of Telehealth and Digital Practice in Physical Therapy” Learning Objectives: Describe the historical perspective of telehealth and digital practice in PT Identify key topics before, during and after a telehealth and digital PT practice Discuss current resources developed by the PT profession (APTA, FSBPT)
Janet Bettger ScD, FAHA	Associate Professor at Duke University; studies implementation and spread of evidence-based interventions, along with team-based research	“To Scale or Not To Scale: Do We Have the Building Blocks to Answer This Question for Telerehabilitation? Learning Objectives: Apply the framework for scaling healthcare interventions to their local context Select outcomes from which to anchor exploration, change or improvement Organize a plan for assessing barriers and using data to reach goals for different stakeholders
Goris Hung, MSc (OT), BSc in Occupational Therapy Jana Cason, DHSc, OTR/L, FAOTA (Co-Presenters)	Has provided OT clinical services for 20 years in pediatrics, geriatrics, and general surgery; early in her career, she trained local therapists and lectured at a medical university in China Professor at Spalding University; Internationally recognized telehealth expert and past chair of the American Telemedicine Association's Telerehabilitation Special Interest Group and American Occupational Therapy Association's Technology Special Interest Section.	“Stepping into a New Era: Fundamentals and Efficacy of Telehealth in Occupational Therapy” Learning Objectives: Define key telehealth terms (telemedicine, telehealth, synchronous, asynchronous, hybrid, originating site and distant site) Identify 3 telehealth practice guidelines, official documents, and resources to guide ethical use of telehealth in OT Identify 3 clinical considerations to improve quality of OT services provided through telehealth Describe 3 evidence-based clinical applications of telehealth in OT
Liz Greenberg, PhD, RN-BC, C-TNP, CNE Ambur Lindstrom-Mette, DNP, RN, FNP-C (Co-Presenters)	Associate Clinical Professor at NAU School of Nursing in Tucson, AZ; Co-authored “The art and science of telephone triage: How to practice nursing over the phone.” Assistant Professor at University of Arizona; board-certified FNP with experience in rural communities, retail clinics, and border health.	Risk Management: Lessons Learned from Telehealth in Nursing Learning Objectives: Recognize the role of the RN and the APRN in telehealth practices Identify areas of risk associated with telehealth services Describe one or more strategies used to reduce risk and help ensure patient safety
All Presenters Facilitated an Interprofessional Breakout Group of Conference Attendees		Interprofessional Case Study: Mr. Doe's Wild Ride

The third session, led by Dr. Alan Lee, focused on advancement of telehealth and digital practice in physical therapy. This session discussed historical perspectives of telehealth and digital practice in physical therapy, and key topics before, during and after telehealth.

Dr. Janet Bettger led the fourth session, focused on outlining the building blocks for scaling up healthcare interventions adapted from a framework used by the Institute for Health Improvement. Strategies for quality improvement, learning health systems, providing equitable care delivery, and conducting implementation research were included.

The fifth and sixth sessions were led by occupational therapists Dr. Jana Cason and Goris Hung. Their presentations provided an overview of evidence-based telehealth service delivery in OT, including identifying practice guidelines and resources to guide implementation of telehealth in OT. Clinical experiences with children and older adults in Hong Kong were described, along with challenges, lessons learned, and opportunities to expand telehealth in OT.

The second day featured three sessions, starting with an overview of risk management in nursing practice from Drs. M. Elizabeth Greenberg and Ambur Lindstrom-Mette. They discussed the roles and contributions of the Registered Nurse (RN) and Advanced Practice Nurse (FNP) in telehealth, possible areas of risk, and risk reduction strategies. Next, workshop participants deliberated a case study from an interprofessional perspective in small breakout groups. The day concluded with a question-and-answer session from all workshop speakers.

## WORKSHOP CONTENT

### SPEECH-LANGUAGE PATHOLOGY SESSION SUMMARY

Medical speech-language pathology has a long history using synchronous and asynchronous telepractice for evaluation and intervention with adults. As early as 1986, Helm-Estabrooks and Ramsberger studied the use of a telephone to supplement in-person therapy for a client with nonfluent, chronic aphasia (Helm-Estabrooks & Ramsberger, 1986). Theodoros (2011) completed a systematic review of the telepractice literature published through 2010 and described changes in the technology used. Some examples included the use of asynchronous videos with telephone therapy sessions (Wilson et al., 2004), videophones (Tindall et al., 2008), computer-based programs (Griffin et al., 2018), and multimedia videoconferencing systems using facility specific platforms (Mashima et al., 2003; Theodoros, 2011). Telepractice studies that were included in the Theodoros review described the emerging evidence in adult neurogenic communication disorders; voice; pediatric speech, language, and literacy disorders; and dysphagia (Theodoros, 2011). Studies that integrated asynchronous, pre-recorded videos were typically used for homework assignments in voice (Griffin et al., 2018) and aphasia (Cherney et al., 2008). Coleman and colleagues (2015) conducted a systematic review of studies that compared telepractice versus in-person evaluation and treatment for cognitive and communication disorders. Ten telepractice group studies were included with adolescent and adult participants with acquired brain injury. The studies in this systematic review focused on motor speech, language, and cognition (Coleman et al., 2015).

In 2015, Molini-Avejonas et al., conducted a systematic review that included 103 research studies on using telepractice in speech-language pathology and audiology. Studies focused on hearing, speech, language, voice, swallowing, multiple disorders (e.g., speech and voice), and other issues (e.g., professional opinions about telepractice). A similar number of telepractice studies were included for assessment and therapy (Molini-Avejonas et al., 2015). More recently, Weidner and Lowman (2020) conducted a systematic review of telepractice-focused articles published between 2014-2019 in speech-language pathology. The authors reviewed 31 studies that focused on screening, evaluation, and treatment for individuals with dysphagia, aphasia, voice, speech, and cognitive deficits (Weidner & Lowman, 2020).

Based on systematic reviews conducted (Coleman et al., 2015; Molini-Avejonas et al., 2015; Theodoros, 2011; Weidner & Lowman, 2020), there is growing evidence to support the use of synchronous and asynchronous telepractice for screening, evaluation, and intervention for a variety of communication disorders. Overall findings indicated little to no significant difference between services administered in-person versus via telepractice. Other strengths identified in the systematic reviews included good intra- and inter-rater reliability when in-person services were compared to telepractice services, cost savings for travel, a decrease in missed caregiver work hours, decreased caregiver burden, similar cost reimbursement as in-person services, and high client, clinician, and family/caregiver satisfaction for telepractice use (Coleman et al., 2015; Molini-Avejonas et al., 2015; Theodoros, 2011; Weidner & Lowman, 2020).

Although many strengths were identified, several limitations were also reported in the telepractice systematic reviews. These included the need to implement better research designs with experimental control groups, improve the description of the participants and severity of communication disorders, better describe the study environments, provide more treatment efficacy data, and consider the variety of evaluations and interventions used (Coleman et al., 2015; Molini-Avejonas et al., 2015; Theodoros, 2011; Weidner & Lowman, 2020). Many of these limitations can be difficult to address due to the heterogeneity and/or multiple communication deficits evident following stroke or traumatic brain injury. Other limitations included the need for better descriptions of the platforms and technology used, more information regarding problems encountered with technology connectivity, and training requirements of participants and caregivers (Theodoros, 2011; Weidner & Lowman, 2020).

Due to COVID-19, there has been a steep learning curve for clinicians using telepractice for service provision. Technological advancements continue to occur rapidly and enhance the ease and quality of providing speech-language pathology services to clients. Clinicians must not only be familiar with the new technology, but they should also ensure that they are providing best practice based on the available research, clinical experience, and client and family preferences. Another consideration is that although clinicians may have the latest technology, clients and family members may not be able to afford similar devices, have adequate WiFi services, or know how to manage technology problems that might occur. Future directions for telepractice research in speech-language pathology should take into consideration these limitations and conduct studies that expand the evidence using synchronous and asynchronous telehealth with various communication disorders.

### PHYSICAL THERAPY SESSION SUMMARY

The American Physical Therapy Association (APTA), the Federation of State Boards of Physical Therapy (FSBPT), and the World Confederation for Physical Therapy (WCPT) (now World Physiotherapy) had a growing interest in telehealth prior to the recent pandemic (COVID-19). In fact, the APTA's past president, Jan Richardson mentioned in her 2000 Presidential Address that physical therapy providers should collaborate with telemedicine providers (Richardson, 2000). Furthermore, the FSBPT developed: (a) a regulation guide for telehealth policy recommendations in April 2015 (https://www.fsbpt.org/Portals/0/documents/free-resources/TelehealthInPhysicalTherapy2015.pdf), and (b) an interstate licensure compact in April 2017 (https://www.fsbpt.org/Free-Resources/Physical-Therapy-Licensure-Compact). Lastly, the WCPT, in collaboration with the International Network of Physiotherapy Regulatory Authorities, developed a task force and report on digital practice in 2019. (See: https://www.fsbpt.org/Portals/0/documents/free-resources/REPORT_OF_THE_WCPTINPTRA_DIGITAL_PHYSICAL_THERAPY_PRACTICE_TASK_FORCE.pdf). This paper utilized the preferred international term “digital practice” in physical therapy, where health care services, support, and information are provided remotely via digital communications and devices (phones, portals, and tablets), with the purpose of facilitating effective delivery of physical therapy services by improving access to care and information and managing health care resources.

For several decades, the telehealth evidence in physical therapy was gaining global momentum (Prvu Bettger et al., 2020). For example, high cost, high resource, in-person physical therapy services for stroke rehabilitation were compared to telehealth physical therapy in randomized trials (Cramer et al., 2019; Prvu Bettger et al., 2020). In these trials, physical therapy telehealth outcomes were similar or better than in-person services with high patient satisfaction. In addition, some trials demonstrated cost saving with home-based telehealth physical therapy. However, earlier systematic reviews opine that evidence alone will not change practice due to the heterogeneous nature of telehealth study designs, and potential research bias and type I error may limit generalizability of telehealth findings.

The recent COVID-19 pandemic provides a lens to the future directions in telehealth physical therapy (Prvu Bettger et al., 2020). On April 30, 2020, the Centers for Medicare and Medicaid Services (CMS) added physical therapy providers in private practice as eligible health care professionals who can furnish and bill for telehealth services, retroactive to March 1, 2020, through the duration of the COVID-19 pandemic. Previously, the CMS added digital practice including e-visits for established patients to interact with a physical therapy provider in secured health portals on March 17, 2020. On May 27 2020, the CMS added outpatient facility-based physical therapy providers to the telehealth provider list. Furthermore, the 2021 CMS Medicare physician fee schedule included a permanent allowance of communication-based technology services (e-visits, virtual check-ins, and remote evaluations of recorded video and images) by physical therapists. This stepwise payment in telehealth and digital physical therapy practice may or may not have encouraged providers and patients to engage in telehealth during the shelter-in-place and stay-at-home orders.

Two APTA surveys (April/May 2020 and July of 2020 (follow-up)) to address the pandemic response provide insights on telehealth utilization from 7,213 physical therapists. Prior to the pandemic, 98% of physical therapists were not providing synchronous telehealth visits. By May 2020, 93% of the school-based systems, 71% of private outpatient offices or group practices, and 69% of academic or postsecondary institutions were adopting telehealth physical therapy services, respectively. By July 2020, 47% of responders had provided live audio and video consults with Zoom (43%), Doxy.me (30%), and other telehealth platforms. The average number of patients seen by telehealth was one to five visits treated per week by 45% of the responders. Although 46% of responders noted equivalent or improved patient satisfaction with telehealth, 54% of responders noted lower patient satisfaction and 53% reported poorer outcomes. Technology barriers were attributed to these findings with 29% of service users lacking adequate technology to access telehealth, and 16% of providers noting a lack of facility technology as a limiting factor. Therefore, technology barriers may require additional training of providers and users in order to advance telehealth and digital physical therapist practice. Lastly, Prvu Bettger et al., (2020) recommend that to advance telehealth, digital practice must address infrastructure, resource, training, and cybersecurity barriers globally. Hence, future advances in physical therapy will require interprofessional practice and rehabilitation collaborations to advance telehealth and digital practice in the digital age.

The progress in telehealth is in part limited by investments made to date into the foundation for its use in standard practice. Access to physical therapy in the United States is already geographically limited with fewer therapists in the southwestern and southeastern rural states (American Physical Therapy Association, 2020). Rural and less populated regions of the United States also face tremendous digital inequities with reduced information technology capacity including weaker broadband internet infrastructure. The “digital divide” is further amplified for populations who do not have access to technology and the digital skills or support to connect with providers for telehealth visits (Chang et al., 2021). Even among therapists and clinical services that rapidly transitioned to telehealth in response to the pandemic, the investment (in broadband, technology, and skills) in populations already at risk for decreased access to care was limited. Addressing digital equity needs to be part of the solution for increasing access to care using telehealth.

Investments are also needed to support physical therapists and the systems in which they work. What happens day-today can be studied to establish real world evidence for optimizing care and outcomes. A learning health system or learning health community is one approach for doing so. Learning health systems are where organizationally aligned infrastructure enables the use of data about care to be integrated with knowledge about processes for care delivery in order to generate new evidence that can be applied back into practice (Agency for Healthcare Research Quality, 2019). This approach to redesigning measurement and evaluation can be particularly useful for overcoming challenges related to acquiring new data. Data often exist as part of care administration but are not integrated to inform daily improvements to practice or to advance the scientific basis for care and outcomes. As more health systems and therapy practices learn and leverage their data, greater attention should be focused on how outcomes related to implementation influence service delivery and client functioning and vice versa. Learning health systems and application of principles from implementation science to telerehabilitation can help establish valuable knowledge for defining what care to provide to achieve which outcomes for which patients.

There was little time at the start of the pandemic to define “a scalable unit” from which to validate and systematically scale and maximize implementation. With in-person care nearly restored to full capacity in countries with lower COVID-19 case rates, health systems, peer groups and professional societies could partner together to document the underlying building blocks to care delivery using telehealth (World Health Organization, 2010). As defined by the WHO, these building blocks include the workforce, service delivery, access to technology, health information systems, financing and leadership and governance. Although, there is likely to be significant variation in service delivery, over time it will be important to determine the assessments and treatments that can be safely and effectively delivered via telehealth. Collaboration across sites and even geographic regions could help define the needs of the workforce and competencies for training. Support will likely be needed for clients to fully participate in care remotely and the therapists' role in this remains unclear. Involvement of health system, regulatory and professional society leadership in these discussions could lead to advances in how technology is used, and the entire therapeutic interaction financed. With clear detail documented on these building blocks, the case for telehealth could be more clearly communicated for wide scale adoption and use.

Critical to communicating scalability will be data on outcomes meaningful to the many stakeholders involved. Consensus on the value proposition for all stakeholders is still in flux and this limits the ability to collectively or locally advocate for greater adoption even when the systems are in place. Although the culture of urgency may have passed, physical therapists play an important role in delineating the benefits to clients, families, themselves as providers, their employers and health insurers. It is critical that providers of care be persistent in communicating benefits, scalability and sustainability. While the pandemic was an unfortunate yet effective accelerator to telehealth use, investment is still needed for measuring the value and underlying building blocks of using telehealth as part of standard of care and examining how this foundation of knowledge can direct improvements to achieving meaningful outcomes for the different stakeholder involved.

### OCCUPATIONAL THERAPY SESSION SUMMARY

Telehealth was first used in rehabilitation in 1998 (Burns et al., 1998). It is becoming increasingly evident that telehealth can improve access to services, prevent unnecessary delays in care (Cason & Jacobs, 2014) and can have similar clinical outcomes to in-person interventions (Kairy et al., 2009). The World Federation of Occupational Therapists in its 2014 telehealth position statement acknowledged telehealth as an “appropriate service delivery model for occupational therapy services when in-person services are not possible, practical, or optimal for delivering care; and/or when service delivery via telerehabilitation is mutually acceptable to the client and provider” (World Federation of Occupational Therapists, 2014, p. 2). Telehealth has been implemented across multiple settings and applications including in early intervention, schools, pediatric private practice, hospitals, productive aging, workplace ergonomics, mental health, and inpatient and outpatient settings in occupational therapy practice (Cason & Jacobs, 2014). In 2018, the American Occupational Therapy Association published a position paper stating that “OT practitioners use telehealth to help clients develop skills; incorporate assistive technology (AT) and adaptive techniques; modify work, home, or school environments; and create health-promoting habits and routines” (AOTA, 2018, p. 2-3).

A systematic review on the effect of telehealth in occupational therapy practice was conducted in 2019 (Hung & Fong, 2019). Fifteen articles published between January 2008 - October 2017 were selected for this review. Applications of telehealth in occupational therapy practice included intervention, training, consultation, education, prevention programs, and the use of assistive technology. Smartphones, telephones, iPads and tablets, videos, internet-based videogames and programs, websites, applications, digital cameras, and emails were used by OT practitioners to provide services synchronously and asynchronously through telehealth. Studies from the review showed significant improvements in functional goals and quality of life, and a significant increase in conducted occupational therapy service. Improvement of pa rticipants' occupational performance, increased carryover of home programs, increased motivation, enhanced home safety, enhanced hand function, improved cognitive function, and decreased parent stress were indicated. The review reported that parents, caregivers, and clients expressed satisfaction with the quality and value of the OT telehealth program. The review concluded that using telehealth in occupational therapy has positive therapeutic effects and is a beneficial alternative service delivery model for varied pathologies, impairment, and age groups. The review also indicated that it is important to provide training to clients, parents, and caregivers prior to OT telehealth intervention, supply necessary tools and/or equipment in advance, and ensure that updated equipment and technical support are available to provide OT telehealth services.

Studies also showed there were many benefits when occupational therapy was provided through telehealth, including high attendance and client compliance (Graham et al., 2013; Kairy et al., 2009; Weidner & Lowman, 2020), intervention content and compatibility were well suited to the client's daily life, costs decreased, and continuity of care improved (Wallisch et al., 2019). Clients who received care via telehealth reported high rates of satisfaction and expressed interest in attending future visits remotely (Cason & Jacobs, 2014; Dirnberger & Waisbren, 2020; Kruse et al., 2017; Li et al., 2020; Powell et al., 2017). Some parents, however, did express their preference to maintain both in-person sessions and telehealth sessions (Johnston, 2019). Also noted, there were factors that may have prevented clients from readily accessing telehealth programs. Older clients, clients with cognitive, intellectual, or psychiatric disabilities (Hermann et al., 2010; Jacobs et al., 2015), those with unstable medical conditions (Salawu et al., 2020), and parents who were reluctant to participate (Johnston, 2019), pointed to the need to continue more traditional in-person therapy approaches that sought to enhance engagement and monitor risk management.

During the COVID-19 pandemic, occupational therapy practitioners began shifting their service delivery model from in-person contact to telehealth. Surveys found that many occupational therapy practitioners reported effective rehabilitation services using telehealth. Their perspective suggests that telehealth could be a permanent service delivery option valued by practitioners and clients after the pandemic (Dahl-Popolizio et al., 2020; Hoel et al., 2021). Still needed, however, is additional policy support and educational training opportunities, along with more widespread access to relevant technology that will help sustain telehealth as a viable adjunct to existing occupational therapy delivery methods. Hoel et al. (2021) also reported that only one-third of occupational therapists conducted assessments through telehealth, and from the systematic review, most assessments used were questionnaires. Hence, more standardized assessments that are amendable to telehealth administration are needed. With the rapid advancement of current technologies, telehealth utilizes internet communication, along with the integration and use of other technological tools, such as home-based sensor monitoring systems and 3D printing, which can support the delivery of tele-evaluation, teleintervention, teleconsultation, and telemonitoring, and can enhance clients' access to care. By working with IT professionals and engineers to improve and develop existing and future technology-based tools, enhanced telehealth sessions will be experienced. More evidence on the efficacy and effectiveness of telehealth in OT practice could be used to advocate for payment and policy that supports use of this service delivery model in occupational therapy.

Telehealth should adhere to evidence-based practices, ethics and equity, enhance quality of care, and empower clients and caregivers. It is important for occupational therapy practitioners to review the latest research and stay current with the evolving trends, knowledge, and technology related to telehealth.

### NURSING SESSION SUMMARY

The COVID-19 pandemic highlighted the value of telehealth nursing, as it did for many virtual practices. Phone calls and messages to nurses increased drastically and nurses worked tirelessly to respond to and assess and direct client callers to the appropriate level of care or to advise those who were sick but could safely stay at home. Nurses were on the front line for those in the community who needed information, reassurance, advice, or even immediate care. Telehealth nurses also remotely monitored employees and patients at-home sick with COVID-19. Using a variety of technologies, telehealth nurses' role during the pandemic included reducing the load on the already strained acute care system through triage, prevention, education, symptom management and even patient outreach (Greenberg, 2022).

The Registered Nurse (RN), educated in telehealth can serve many roles (Rutledge & Gustin, 2021). The role of the RN includes client care management and coordination of services. When symptoms are present, the RN can assess and triage, and advise clients, that is, identify the health need, the level of urgency, and the appropriate level of care. RN's act as client advocate; they assess, record, and communicate the situational and individual factors that affect the health status of the client in order to provide truly individualized care. Competencies in these areas are clearly described in the profession's s tandards of care (American Nurses Association, 2018, 2021), the code of ethics (American Nurses Association, 2015), and individual nurse state practice acts.

As a member of the healthcare team, interprofessional practice (IPP) is a natural and necessary part of nursing. To help facilitate teamwork, and prevent delays or gaps in care, competence in communication, collaboration, and care coordination are expectations of the RN whether care is delivered in-person or via telehealth (American Nurses Association, 2018, 2021). Even though nurses are not always part of the telehealth team, educational opportunities are needed to ensure that they are aware of, and can meet, the expectations of their license and education when practicing in the telehealth arena (Schweickert & Rutledge, 2020).

The Advanced Practice Registered Nurse (APRN) must meet the competencies and responsibilities of the RN but the APRN has an expanded scope of practice. In those states where they are granted practice privileges and prescriptive authority, the APRN, usually a Nurse Practitioner (NP) can act as an independent provider. The NP, as an independent practitioner, plays a key role in telehealth, including the development, implementation, and oversight of new telehealth delivery models (Schweickert & Rutledge, 2020).

From the telephone to video to wearable devices and apps, technology is being integrated into nursing practice. And even though clients and providers are satisfied with telehealth, there are still risks involved. Taking the time to get to know and form a relationship with the client is critical. Clients who feel known, heard, and supported are more inclined to engage in treatment and less likely to file a lawsuit. Telehealth lawsuits are not uncommon. They have primarily focused on errors in communication, diagnosis, and equipment failures. The following client centered lessons learned can be useful for all telehealth providers and clinicians in managing the risks associated with the use of telehealth.

- Communication:
  - Stop, listen, and follow-up on clients' comments, concerns, or complaints.
  - Assess literacy level, vision, and hearing, and communicate accordingly.
  - Access and use an interpreter or interpreter service (e.g., LanguageLine®) if a language barrier exists.
  - Document findings for other members of the interprofessional team.
- Assessment:
  - Assess and document accurately, the client conditions, interventions, and outcomes.
  - Assess equipment as well as the client's use to ensure safety.
  - Assess for cultural differences and adapt care accordingly.
  - If client has a new or worsening complaint, the nature and urgency of the complaint should be assessed and documented by qualified professional.
- Documentation:
  - Must be timely and accurate to be useful to all team members.
  - Document thoroughly to illustrate events and status.
  - Document evidence of the intervention, the immediate outcome, and the next step(s).

Effective communication between and among those involved, accurate and appropriate assessments, and timely and thorough documentation are important strategies in reducing the risks associated with care delivery using technology. Attending to issues of scope of practice and organizational policy and procedures is also important.

### USE OF A CASE STUDY TO ILLUSTRATE INTERPROFESSIONAL EDUCATION AND PRACTICE

A case study developed by Britton, Sprang and Tracy and presented at the MidAtlantic Resource Center (MATRC) annual conference on March 31, 2014 was used with MATRC permission. The title of the case study was “Mr. Doe's Wild Ride,” and it profiled a 71-year-old married retired male who is living independently. His health conditions included that he is a smoker, has non-insulin dependent diabetes, high blood pressure, chronic low back pain, arthritis, frequent headaches and a family history of cancer, heart, and lung problems. The case study starts with Mr. Doe not feeling well, so he schedules a visit with his primary care provider (PCP), and lab work is completed. His PCP prescribes blood pressure medication and discusses lifestyle changes that would help his health conditions. When Mr. Doe gets home, he does not react well to the blood pressure medication and loses interest in his treatment recommendations, so he stops taking his medicine. After a few weeks, Mr. Doe collapses at home and his wife calls 911. After he is released from the hospital, he continues to ignore recommendations for improving his health, and he goes through a continuous cycle of acute incidents, and visits to the clinic or hospital. (For more information on the case study, see: https://www.facebook.com/MATRC/videos/624584960938024/).

To enhance interprofessional practice among all disciplines represented at the conference, some modifications were made. The case study was provided in advance of the conference and introduced to workshop participants. Participants were then divided into breakout sessions for further discussions regarding the case. Groups consisted of 6-8 members from various disciplines who analyzed the case from the perspective of their specialty areas and what each discipline could contribute in interprofessional practice (IPP). Pre-selected group facilitators led discussions and had pre-determined questions to enhance and promote discussion. Following the breakout groups, all participants reconvened, and facilitators shared group responses to the questions. Additional questions were asked during the reconvening for further discussion and IPP considerations. Specifically, discussion following the case study review focused primarily on missed opportunities. Each of the disciplines identified areas for improvement. Perhaps the biggest take-away was the general agreement among participants that the opportune time to identify patient needs and get a team together is at discharge from acute care.

### BEST PRACTICES, FUTURE IMPLICATIONS, AND RECOMMENDATIONS FOR INTERPROFESSIONAL PRACTICE IN TELEHEALTH

The Institute of Medicine identified core competencies needed for healthcare professionals participating in IPP (Institute of Medicine Committee on Quality of Health Care, 2001; Institute of Medicine Committee on Health Professions Education, 2003). When providing IPP, not only is professional competence in each discipline necessary, but also cultural competence for the clients who are served. Healthcare professionals must be willing to work in interdisciplinary teams that require collaboration, education, and training. The key to working as a cohesive team requires coordination, cooperation, and open communication. Although most professionals report that they work with a variety of healthcare professionals, knowledge about IPP and whether actual IPP are truly evident in the services provided remains unclear at least among rehabilitation professions.

Evidence-based practice should not only be employed by each profession, but also as it relates to IPP. This is especially true for telehealth IPP when healthcare professionals need to consider the technology, platform and equipment used, and ensure that compliance is met (Institute of Medicine Committee on Quality of Health Care, 2001; Institute of Medicine Committee on Health Professions Education, 2003). Additionally, service provider equipment (microphone, video camera), number of clients and providers, and client's equipment must be taken into consideration (Institute of Medicine Committee on Quality of Health Care, 2001; Institute of Medicine Committee on Health Professions Education, 2003). Other important aspects of telehealth IPP include determining which team members are essential for the care of the client and identifying a lead therapist based on client need.

Overall, IPP has been shown to improve attitudes, perceptions, and clarity in roles of other disciplines, collaborative knowledge and skills, communication, and teamwork (Lim & Noble-Jones, 2018; Maheu et al., 2018; Reeves, 2016). Due to the need for synchronous online services during the pandemic, more research is expected in the efficacy and use of telehealth IPP.

## CONCLUSIONS

This workshop discussed telehealth terminology and evolution, and it provided opportunities for SLPs, OTs, PTs and nurses to discuss discipline-specific and interprofessional best practices in telehealth. In addition, a case study was discussed in interprofessional breakout groups to establish unique and overlapping areas of practice. Each discipline established the need for more formal education and more IPE and IPP to optimize telehealth. Formal training in telehealth best practices and training for all collaborators will help minimize challenges as telehealth continues to grow and contribute to health care in the future.
